# Phase-dependent asymmetry of pattern masking in natural images explained by intrinsic position uncertainty

**DOI:** 10.1167/jov.23.10.16

**Published:** 2023-09-25

**Authors:** Anqi Zhang, Eric S. Seemiller, Wilson S. Geisler

**Affiliations:** 1 Center for Perceptual Systems, University of Texas at Austin, Austin, TX, USA; 2 Department of Physics, University of Texas at Austin, Austin, TX, USA; 3 711th Human Performance Wing, Air Force Research Labs, Wright-Patterson AFB, OH, USA; 4 Center for Perceptual Systems, University of Texas at Austin, Austin, TX, USA; 5 Department of Psychology, University of Texas at Austin, Austin, TX, USA

**Keywords:** pattern masking, intrinsic uncertainty, similarity, detection

## Abstract

A number of recent studies have been directed at measuring and modeling detection of targets at specific locations in natural backgrounds, a key subtask of visual search in natural environments. A useful approach is to bin natural background patches into joint histograms with bins along specific background dimensions. By measuring psychometric functions in a sparse subset of these bins, it is possible to estimate how the included dimensions jointly affect detectability over the whole space of natural backgrounds. In previous studies, we found that threshold is proportional to the product of the background luminance, contrast, and similarity; a result predicted by a simple template-matching observer with divisive normalization along each of the dimensions. The measure of similarity was the cosine similarity of the amplitude spectra of the target and background (*S_A_*)—a phase-invariant measure. Here, we investigated the effect of the cosine similarity of the target and background images (*S_I|A_*)—a phase-dependent measure. We found that threshold decreases monotonically with *S_I|A_* in agreement with a recent study (Rideaux et al., 2022). In contrast, the template-matching observer predicts threshold to be a U-shaped function of *S_I|A_* reaching a minimum when the target and background are orthogonal (*S_I|A_* = 0). Surprisingly, when the template-matching observer includes a small amount of intrinsic position uncertainty (measured in a separate experiment) the pattern of thresholds is explained.

## Introduction

Neural computations in visual systems evolve to exploit the statistical properties of natural stimuli that are useful for performance of the organism's natural tasks. Thus, to understand and predict visual behavior, it is important to measure and analyze the statistical properties of natural stimuli, and to determine the computational principles relevant for performance of the organism's natural tasks, given its natural stimuli. Such analyses can produce principled hypotheses that can be tested either with naturalistic or artificial stimuli.

One of the most common natural tasks for humans is detection of known objects in the images the eye captures from the environment. There has been considerable effort directed at understanding how the statistical properties of natural images affect human ability to detect targets in natural backgrounds. However, most of the early studies either tested only a small number of natural stimuli (Caelli & Moraglia, 1986; Rohaly, Ahumada, & Watson, 1997; Chandler, Gaubatz, & Hemami, 2009), tested natural stimuli with altered statistical properties (Bradley, Abrams, & Geisler, 2014), or used multiple-interval forced choice procedures (Caelli & Morglia, 1986; Chandler et al., 2009; Alam, Vilankar, Field, & Chandler, 2014), which are not representative of detection under natural conditions where one typically does not get to compare the exact same image with and without the target.

More recently, Sebastian, Abrams, and Geisler (2017) and Sebastian, Seemiller, and Geisler (2020) used a constrained sampling approach to measure how various properties of natural-image backgrounds affect detection performance in a simple yes/no task with stimulus durations corresponding to a typical duration of single fixations in visual search (250 ms). Specifically, Sebastian et al. (2017) binned millions of patches of natural background (the size of the detection target) into joint histograms along several dimensions: luminance (*L*), RMS contrast (*C*), and cosine similarity of the target and background amplitude spectra (*S_A_*), a phase-invariant similarity measure. The histograms contained 1000 bins (10 along each dimension). They then measured thresholds for backgrounds sampled from a sparse subset of bins across the whole space. For windowed sinewave and plaid targets added to the backgrounds, they found that amplitude threshold *a_t_* increases linearly along all three dimensions, consistent with separable Weber's law: *a_t_* ∝ *L* × *C* × *S_A_*. The separability finding was subsequently confirmed by Dorronsoro, Walshe, Sebastian, and Geisler (2018). Sebastian et al. (2020) found another factor, the partial-masking factor ‖*T_P_*‖, that multiplicatively combines with the other factors: *a_t_* ∝ *L* × *C* × *S_A_*/‖*T_P_*‖. The partial-masking factor captures the fact that for natural backgrounds having the same luminance, contrast, and phase-invariant similarity, the more the luminance and contrast of the background varies within the target region the easier to detect targets (for more details see Sebastian et al., 2020; and Zhang & Geisler, 2022).

Recently, Rideaux et al. (2022) measured the effect of similarity in the spatial domain, which is a phase-dependent statistic. They measured the detectability of derivative-of-Gaussian targets that were added in different phases with respect to contours located within natural images. They found that thresholds were lowest when the target was in phase with the contour and highest when 180° out of phase. In other words, threshold decreased as a function of phase-dependent similarity, just the opposite effect reported by Sebastian et al. (2017) and Sebastian et al. (2020) for phase-invariant similarity. Rideaux et al. (2022) also found that the effects of phase-dependent similarity dominate the effects of phase-invariant similarity. However, their task was not a simple detection task but covert visual search, their stimuli were selected by a highly constrained procedure, and their natural backgrounds were object-dominated, often indoor, scenes rather than outdoor natural scenes like those of Sebastian et al. (2017) and Sebastian et al. (2020).

Here we describe an experiment measuring the effects of phase-dependent similarity for the natural stimuli in the simple detection task of Sebastian et al. (2017) and Sebastian et al. (2020). Like Rideaux et al. (2022), we found that the effect of phase-dependent similarity is highly asymmetric. However, unlike Rideaux et al. (2022) we found that phase-independent similarity and phase-dependent similarity are both major factors affecting detection accuracy in natural backgrounds.

Interestingly, when we applied the normalized template-matching observer of Sebastian et al. (2017) and Sebastian et al. (2020) to our stimuli, we found that it predicts symmetric thresholds as a function of phase-dependent similarity, reaching the minimum when the target and background are orthogonal (phase = 90°). This prediction holds independent of the level of phase-independent similarity. However, the template-matching observer did not include intrinsic position uncertainty, which is known to exist in the human visual system. To evaluate the potential role of intrinsic position uncertainty, we directly measured the level of position uncertainty in our observers in a separate position-discrimination experiment for the same target and the same background diameter used in the masking experiment. Surprisingly, we found that if this small amount of intrinsic position uncertainty is included in the template-matching observer, then it predicts an asymmetric pattern of thresholds similar to that of the human observers. We conclude that intrinsic position uncertainty, and the additional extrinsic position uncertainty in the study by Rideaux et al. (2022), provide a plausible explanation of the asymmetric masking functions reported in both studies.

## Methods

All experimental procedures were approved by the University of Texas Institutional Review Board and informed consent was obtained from all participants.

### Experiment 1: Amplitude-spectrum similarity and image similarity

**Figure 1.**
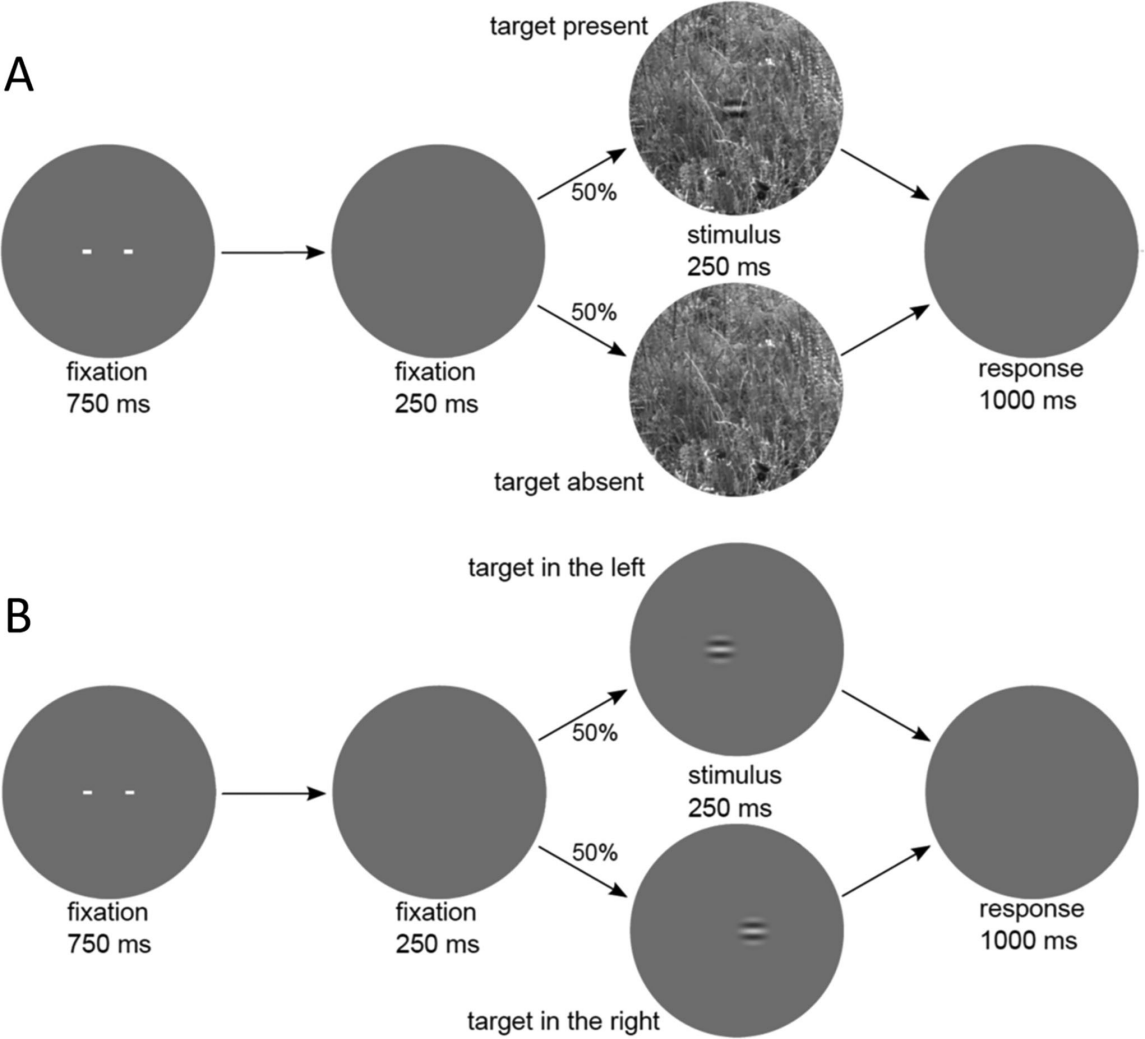
Timeline and task for two experiments. (**A**) Masking experiment. The task is to report whether the wavelet target is present or absent. When present, the target is always in the center of the background region. (**B**) Position-discrimination experiment. The task is to report whether the wavelet target is left or right of the center of the background region.

Psychometric functions were measured in a yes/no detection task, as a function of the amplitude of a horizontal 4-cpd raised-cosine windowed sinewave (wavelet) target in cosine phase (see Figure 1A). On each trial, a natural background was randomly sampled (without replacement) from a large set of natural backgrounds having two levels of amplitude-spectrum similarity *S_A_* and five levels of image similarity *S*_*I*|*A*_. On half the trials the target was added to the center of the background. To obtain the backgrounds, we started with a large set of calibrated, high-resolution (4284 × 2844), 14-bits per color natural images that were converted to grayscale and cropped into background patches the size of the target. These background patches were then sorted into a three-dimensional histogram having 10 levels of luminance, 10 levels of RMS contrast, and 10 levels of similarity between the amplitude spectrum of the background and target (for more details see Sebastian et al., 2017 and https://natural-scenes.cps.utexas.edu). In the present experiment, all the backgrounds were sampled without replacement from bins having the same level of luminance and contrast, and two different levels of amplitude-spectrum similarity. For each level of amplitude-spectrum similarity the backgrounds were sorted into five bins (quintiles) of image similarity.

Amplitude-spectrum and image similarity are defined on the mean-subtracted target and background (i.e., the target and background are each set to have a mean of zero). The amplitude-spectrum similarity between the target and background was defined to be the cosine similarity between the amplitude spectrum of the mean-subtracted target and background (the dot product of the two spectra divided by the product of their Euclidean norms)

(1) $$\begin{array}{c} S_{A}=\frac{A_{t}}{\left|\right|A_{t}\left|\right|}\cdot \frac{A_{B}}{\left|\right|A_{B}\left|\right|}\; \end{array}$$

The image similarity between target and background was defined to be the cosine similarity between the mean-subtracted target and background:

(2) $$\begin{array}{c} S_{I|A}=\frac{I_{t}}{\left|\right|I_{t}\left|\right|}\cdot \frac{I_{B}}{\left|\right|I_{B}\left|\right|}\; \end{array}$$

We use “|A” in the subscript to emphasize that this cosine similarity was computed conditional on the value of *S_A_*, even though the formula (Equation 2) does not directly depend on *S_A_*. The two levels of amplitude-spectrum similarity were 0.18 (low similarity) and 0.38 (high similarity). These two levels correspond to the second and ninth bin of the 10 bins in Sebastian et al. (2017). They were picked to be near the ends of the range in natural images, yet to have a large number of image patches. The five levels of image similarity were defined to be the midpoint of the quintiles of image similarity within each of the two amplitude-spectrum similarity bins (−0.15, −0.06, 0.00, 0.06, 0.15 for the high amplitude similarity bin, and −0.05, −0.02, 0.00, 0.02, 0.05 for the low-amplitude similarity bin).

At the beginning of each trial, the central fixation cue was displayed for 750 ms and was then extinguished for 250 ms. Next, the stimulus was displayed for 250 ms, followed by a response interval of one second. Feedback was given on each trial. The displayed background patches had a diameter of 516 pixels (4.3°) and, hence, included the context region surrounding the smaller 96-pixel diameter background patch (0.8°) used to determine the luminance, contrast and similarities in the target region. The stimuli were displayed at 120 pixels/deg, on a Sony GDM-FW900 CRT Monitor with a total background size of 19.2° × 12°. The luminance of the screen outside the background patch was set to the mean luminance of the background patches, which was always 50 cd/m^2^. The target was present on half of the trials. When present, the target appeared in the center of the background. The amplitude *a* of the target was defined to be the square root of the sum of the squared pixel values (the square root of the target energy). For plotting convenience, we divided the actual RMS amplitude by 97.8; thus an amplitude of 1 in the plots corresponds to 97.8.

**Figure 2.**
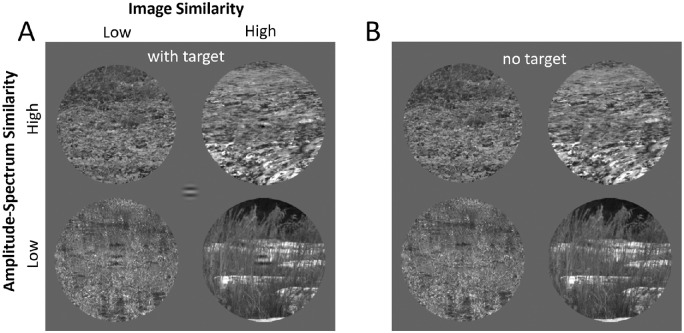
Masking by amplitude-spectrum similarity and image similarity. (**A**) Examples of stimuli with the 4 cpd wavelet target present. (**B**) The same example backgrounds with target absent. The target tends to be harder to see when the amplitude-spectrum similarity of the target and background is high and when the image similarity of the target and background is low (given the same amplitude-spectrum similarity).

For presentation, the 14-bit gray-scale images were clipped to the upper ninety-ninth percentile gray level, gamma-compressed based on the measured gamma of the monitor, and then quantized to the range of 0–255 gray levels. The linear relationship between the desired and displayed luminance was verified with a photodiode following the calibration procedure. Figure 2 shows examples of the stimuli from the two levels of amplitude spectrum similarity and the two extreme levels of image similarity (first and fifth quintiles).

Psychometric functions were measured on three human observers who all had normal or corrected-to-normal spatial vision. The observer's head was stabilized with a chin and head rest. For each amplitude-spectrum similarity there were 2000 trials spread over four sessions (10 target amplitudes × 50 trials × 4 sessions = 400 trials per image similarity quintile). Target amplitude and amplitude-spectrum similarity were blocked, whereas image similarity was unblocked. For each target amplitude within a quintile, the numbers of hits and correct rejections were converted to a value of discriminability $d^{'}$ and criterion γ, using the standard formulas from signal-detection theory. The values of $d^{'}$ were then converted to a value of *PC*_max_ (the percent correct if the criterion were placed optimally) using the standard formula PCmax=Φ(d'/d'22). Finally, thresholds were estimated by maximum-likelihood fitting the values of *PC*_max_ with a generalized cumulative Gaussian function:

(3) $$\begin{array}{c} PC_{\max}\left(a\left|\alpha ,\beta \right.\right)=\Phi \left[\frac{1}{2}{\left(\frac{a}{\alpha }\right)}^{\beta }\right]\; \end{array}$$

We define threshold to be the target's RMS amplitude *a* where *d*′ = 1, and thus the estimated threshold is simply the estimated value of α.

### Experiment 2: Intrinsic position uncertainty

The purpose of Experiment 2 was to measure the intrinsic position uncertainty in two of our observers. The target, the background diameter, and the trial sequence were identical to those in the masking experiment (see Figure 1B). However, the background was uniform gray with a thin black line marking the border, the target was clearly visible (RMS contrast = 4%), and the task was to report whether the target was shifted to the left or right of the center of the circular background region. Psychometric functions were measured for 10 levels of target offset (30 trials/level × 4 sessions = 120 trials/level). As in the masking experiment, the discriminability and criterion were estimated for each target offset level, and then the discriminability was converted to *PC*_max_. The probability distribution of intrinsic position uncertainty is unknown, and, hence, we considered two possible distribution shapes: two-dimensional Gaussian with standard deviation σ, and two-dimensional uniform with radius ρ. The shape of the predicted psychometric function is completely determined by the single parameter of the assumed distribution shape, and hence the parameter for each assumed shape was estimated by finding the maximum likelihood fit to the psychometric data.

#### Model observers

To help understand human performance, we simulated the performance of several model observers. The first was a simple template-matching (TM) observer that computes on each trial the normalized response of a template (receptive field) that matches the shape of the target,

(4) $$\begin{array}{c} R_{TM}^{'}\left(x_{0}\right)=\frac{1}{\sigma }\sum_{x}t\left(x-x_{0}\right)I\left(x\right)\; \end{array}$$

where *t* is the target shape, *I* is the image, **x** = (*x*, *y*) represents a spatial coordinate, **x**_0_ represents the location of the target (when present), and σ is the standard deviation of the unnormalized template response. If the template response exceeds a decision criterion γ the observer makes the behavioral response “present”, otherwise “absent”. The TM observer is the ideal observer for detecting additive targets in white noise (Green & Swets, 1966; Burgess, Wagner, Jennings, & Barlow, 1981). It provides a useful performance benchmark because it captures much of the detection information (but not all, because of the correlational structure of natural images).

The second model observer is an eye-filtered template-matching (ETM) observer that takes into account the human contrast sensitivity function,

(5) $$\begin{array}{c} R_{\text{ETM}}^{'}\left(x_{0}\right)=\frac{1}{\sigma_{E}}\sum_{x}t_{E}\left(x-x_{0}\right)I_{E}\left(x\right)\; \end{array}$$

where *I_E_* is the input image filtered by the human CSF and *t_E_* is the target shape filtered by the human CSF (e.g., see Burgess, 2011), and σ_*E*_ is the standard deviation of the unnormalized eye-filtered template response. Here we use the ModelFest CSF (Watson & Ahumada, 2005; see Zhang & Geisler, 2022, for more details).

The third model observer is an ETM observer with intrinsic position uncertainty (the UETM observer). The simplest UETM observer uses the so-called “max rule”—compute the maximum template response after weighting by the uncertainty distribution (prior) over target location:

(6) $$\begin{array}{ccc} & & \;R_{\text{UETM}}^{'}=\max_{x}\left[d^{'}{R^{'}}_{\text{ETM}}\left(x\right)+\ln p_{U}\left(x-x_{0}\right)\right]\; \end{array}$$

where *p_U_*(**x**) is the uncertainty distribution, and d'=a/aσσE. This max response is then compared to the decision criterion. Detecting a target when there is intrinsic location uncertainty is effectively a visual search task with intrinsic rather than extrinsic uncertainty. It can be shown that the max rule is the optimal search strategy when there are independent potential target locations and when the task is to indicate either the target location or that the target is absent (e.g., Walshe & Geisler 2022). In the real world, useful visual search usually involves identifying the target's location (if present), and thus the max rule in Equation 6 is arguably the most practical rule for visual systems to adopt. However, if the task is instead to simply indicate whether the target is present or absent, then the optimal rule is summation over the possible target locations:

(7) $$\begin{array}{c} R_{\text{UETM}}^{'}=\sum_{x}\exp\left[d^{'}{R^{'}}_{\text{ETM}}\left(x\right)\right]p_{U}\left(x-x_{0}\right)\; \end{array}$$

Strictly speaking, the current task is to indicate whether the target is present or absent, not its apparent location. Thus it is possible that the visual system uses the sum rule under such circumstances. Given this possibility, we consider both versions of the UETM observer. Equations 6 and 7 are for the case where the template responses are Gaussian distributed, which they are, to good approximation, for our targets and backgrounds (Sebastian et al., 2017). In the Appendix, we also derive the max and sum rules for arbitrary probability distributions.

In addition to these model observers, we considered versions where the template responses are a mixture of simple and complex template responses. The simple template responses are given by Equations 4 and 5. A complex template combines the response of the simple template *t* with the response of the same simple template with its phase spectrum shifted by 90° *t*_⊥_. Thus the complex template response corresponding to Equation 4 is

(8) $$\begin{array}{ccc} & & \;R_{\text{CTM}}^{'}\left(x_{0}\right)\\ & & \;\;\;\;=\frac{1}{\sigma }\sqrt{{\left[\sum_{x}t\left(x-x_{0}\right)I\left(x\right)\right]}^{2}+{\left[\sum_{x}t_{⊥}\left(x-x_{0}\right)I\left(x\right)\right]}^{2}}\; \end{array}$$

For the target in our experiments the phase-shifted template is a wavelet in sine phase rather than cosine phase. In other words, Equation 8 gives the response of a canonical complex cell with orientation and spatial frequency tuning matched to the target (Adelson & Bergen, 1985). Template responses that are a mixture of simple and complex template responses are a weighted sum of the two template responses. The mixture response corresponding to Equation 4 is

(9) $$\begin{array}{c} R_{\text{MTM}}^{'}\left(x_{0}\right)=\alpha R_{\text{TM}}^{'}\left(x_{0}\right)+\left(1-\alpha \right)R_{\text{CTM}}^{'}\left(x_{0}\right)\; \end{array}$$

where α is the mixture parameter that can vary between 1 (just the simple template) and 0 (just the complex template). We decided to also consider such mixture responses, because many of the neurons in primary visual cortex of primates are complex cells, and hence detection in humans may involve pooling responses from both kinds of cells.

The simulated behavioral responses of all the model observers were analyzed in the same way as those of the human observers. For each background condition and target amplitude, the value of $d^{'}$ was computed and converted to a value of *PC*_max_. Thresholds were then estimated by maximum-likelihood fitting the values of *PC*_max_ with a generalized cumulative Gaussian function.

## Results

### Experiment 1: Amplitude-spectrum and spatial similarity

**Figure 3.**
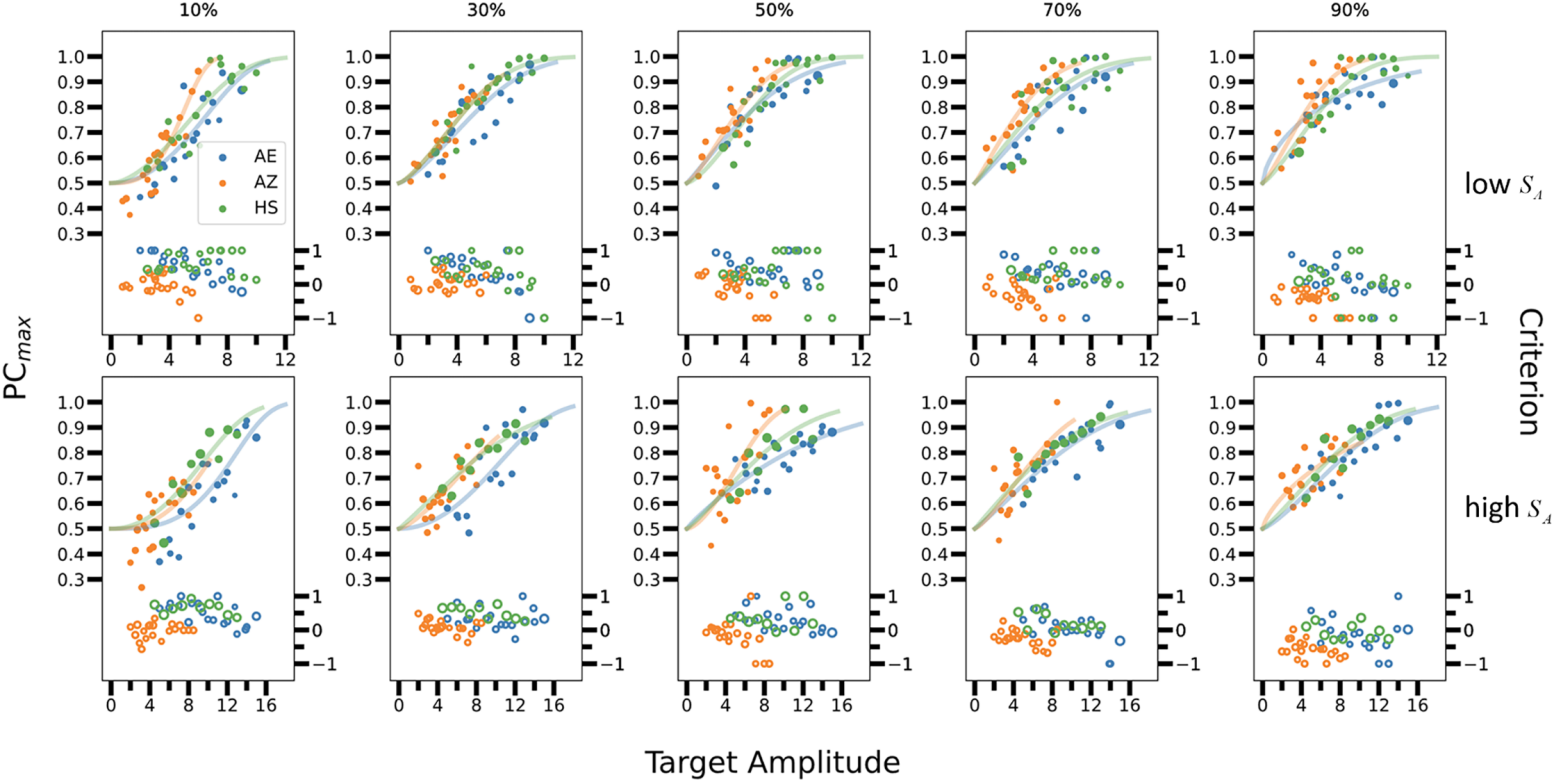
Psychometric functions from Experiment 1. Estimated bias-corrected percent correct (left axis) and criteria in units of standard deviation (right axis) for three observers as a function of target amplitude. The upper row of plots is for the low phase-invariant similarity (*S_A_*), the lower row the high phase-invariant similarity. The plots within each row are for different percentiles of phase-dependent similarity (*S_I|A_*).

The psychometric data for the three observers in Experiment 1 are shown in Figure 3. As can be seen, the data are similar for all three observers, with roughly monotonic psychometric functions and relatively constant decision criteria. One interesting feature that we will come back to later is that for low target amplitudes, the bias corrected proportion correct *PC*_max_ is systematically below 0.5, which corresponds to a negative detectability *d*′.

**Figure 4.**
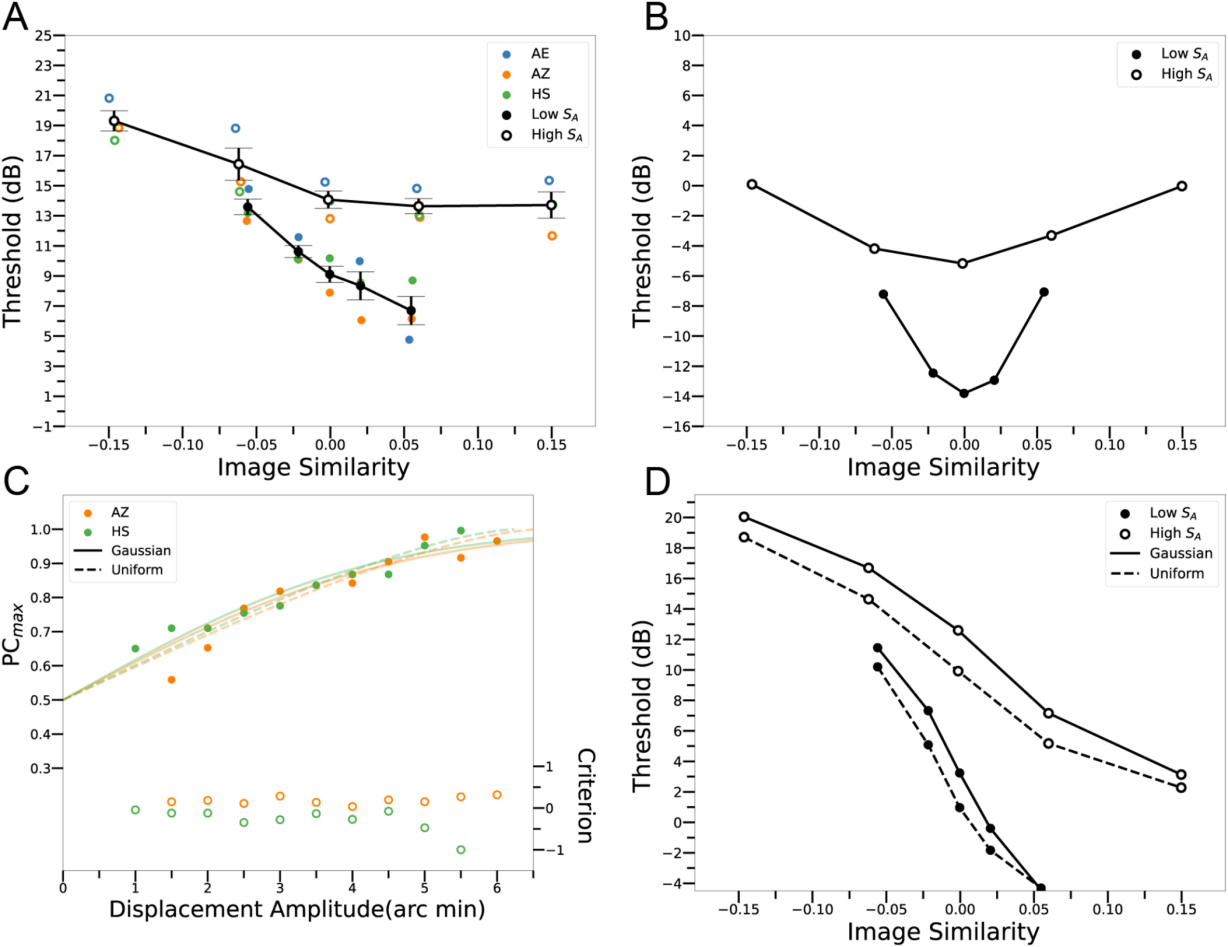
Measurements and predictions. (**A**) Amplitude thresholds for a wavelet target in natural backgrounds, as a function of the image similarity *S_I|A_* of the target and background, for two levels of amplitude-spectrum similarity *S_A_* of the target and background. (**B**) Amplitude thresholds of a template-matching observer (ETM) that prefilters the input image with the average human eye filter (human CSF). The values of the standard deviation σ_*E*_ in Equation 5 was 250.2 for the high similarity condition and 91.5 for the low similarity condition. (**C**) Psychometric functions of two observers in the position-discrimination experiment (Experiment 2). The solid symbols (left axis) are proportion correct after correcting for the estimated bias (right axis). Defining threshold as a *d*′of 1.0, the discrimination thresholds are approximately ± 2 arcmin. If the intrinsic position noise is Gaussian, this 2 arcmin threshold corresponds to a distribution with a standard deviation of 3.4 arcmin. If the intrinsic position noise has a uniform distribution, the threshold corresponds to a distribution with a radius of 6.5 arcmin. (**D**) Amplitude thresholds of a UETM observer that prefilters the input image with the average human eye filter and has the intrinsic position noise estimated from the human observers in Experiment 2, assuming a Gaussian or a uniform noise distribution.

The measured thresholds in Experiment 1 are shown in Figure 4A, which plots amplitude threshold in decibels (dB = 20log_10_(*a*)) as function of image similarity for the low (0.18) and high (0.38) amplitude-spectrum similarity conditions. The colored symbols are thresholds for the individual observers and the black symbols are the average thresholds. As can be seen, the thresholds decline monotonically with image similarity and are higher when the amplitude-spectrum similarity is high. All three observers show a similar trend (the error bars are ±1 standard error across the observers).

**Figure A1. figA1:**
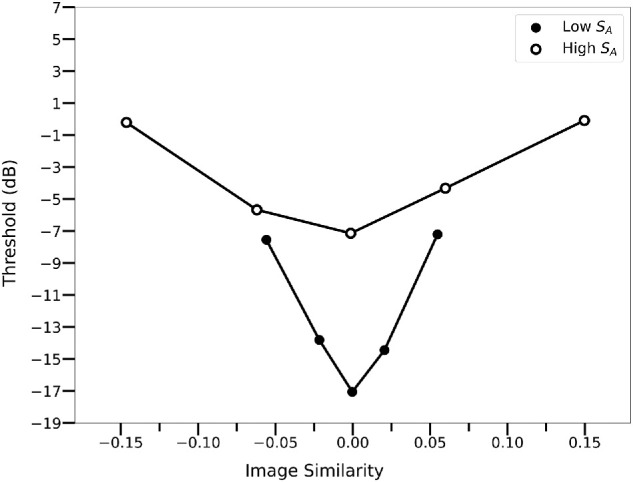
Threshold of the template matching (TM) observer for the stimuli in the current study. The pattern of thresholds is qualitatively similar to that of the ETM observer (compare with Figure 4B).

Figure 4B shows the thresholds of the template-matching observer that prefilters input images with a filter matching the shape of the ModelFest CSF (the ETM observer, Equation 5). Rather than declining monotonically with image similarity, the thresholds follow symmetrical U-shaped functions with minima at an image similarity of 0.0 (i.e., when the target and background are approximately orthogonal). The simpler TM observer (Equation 4) has qualitatively similar pattern of thresholds (see Appendix Figure A1).

The U-shaped function with image similarity is intuitive because the template response to the target stays the same independent of the orthogonality of the background, whereas the template response to the background is weaker when the background is more orthogonal to the target. As expected, when the background responses are weaker, they are also less variable, and hence the signal-to-noise ratio is higher. Clearly, human behavior is qualitatively very different from that of a template-matching observer.

Template matching observers capture most of the information in simple detection experiments, and hence the highly asymmetric pattern of human thresholds represents an unusually strong qualitative deviation from the hypothesis of uniform information loss (constant efficiency). One important biological factor that the ETM observer does not incorporate is intrinsic position uncertainty (e.g., Nolte & Jaarsma, 1967; Swensson & Judy, 1981; Shaw, 1982; Pelli, 1985; Michel & Geisler, 2011; Geisler, 2018). Position uncertainty often does not change the qualitative pattern of predicted thresholds. For example, it is not expected to change the effect of amplitude spectrum similarity on detection thresholds (Walshe & Geisler, 2020). However, it could still change the effect of image similarity. To explore this possibility, we directly estimated the intrinsic position uncertainty of two of our observers for the same target, background diameter, and trial sequence as the masking experiment.

### Experiment 2: Intrinsic position uncertainty

Figure 4C shows the bias-corrected percent correct as a function of the target offset in the location discrimination task, for two subjects. The solid circles show bias-corrected percent correct, and the open circles show criterion bias as a function of target offset from the center of the background region (see Figure 2B). The percentage correct increases monotonically with target offset, and the criterion is relatively constant, and the bias is relatively small. The solid blue and orange curves show the maximum likelihood fit to each subject assuming a Gaussian intrinsic-uncertainty distribution and the dashed curves assuming a uniform intrinsic-uncertainty distribution. As can be seen, the maximum-likelihood fits using the Gaussian and uniform distributions are about equally good, and, hence, the psychometric data does not discriminate between them, or presumably between any intermediate distributions. The estimated Gaussian standard deviation is 3.4 arcmin and the estimated uniform radius is 6.5 arcmin. In both cases, these parameters correspond to a position threshold of around ±2 arcmin, which is consistent with previous measures of position discrimination thresholds under similar conditions (e.g., the “bullseye” thresholds reported in Legge & Campbell, 1981). Note that the level of position uncertainty varies depending on the size and shape of the reference frame. For example, if the size of the background region were smaller than 4.3^o^ the position thresholds (and uncertainty) would be smaller.

#### Uncertain template matching observers

Figure 4D shows the performance of the UETM observer using the max rule (Equation 6) for the Gaussian and uniform uncertainty distributions, with the parameter values estimated in Experiment 2. These predictions are essentially parameter free. The predictions for the Gaussian and uniform distributions are very similar and are qualitatively similar to human performance shown in Figure 4A. In other words, an intrinsic location uncertainty corresponding to the human location discrimination threshold of just ± 2 arcmin explains the highly asymmetric effect of spatial image similarity (*S*_*I*|*A*_) on human detection thresholds.

Comparison of Figures 4B and 4D shows, as expected, that the UETM observer's overall thresholds are substantially higher (about 20 dB; i.e., an order of magnitude) than those of the ETM observer and that the predicted average difference in thresholds (in dB) between low and high amplitude spectrum similarity (*S_A_*) remains about the same.

The asymmetric effect of image similarity when there is intrinsic uncertainty makes intuitive sense. When the background is in phase with the target (e.g., percentile = 90%), then the effect of the intrinsic uncertainty is reduced because the max will tend to be at or near the location of the target, and this benefit equals or outweighs the reduced variance of orthogonal backgrounds (percentile = 50%). Similarly, when the background is out of phase with the target (e.g., percentile = 10%), the max template responses will tend to be away from the target location when the target amplitude is low and, hence, be dominated by background noise and will only shift toward the actual target location as the target amplitude is increased.

**Figure A2. figA2:**
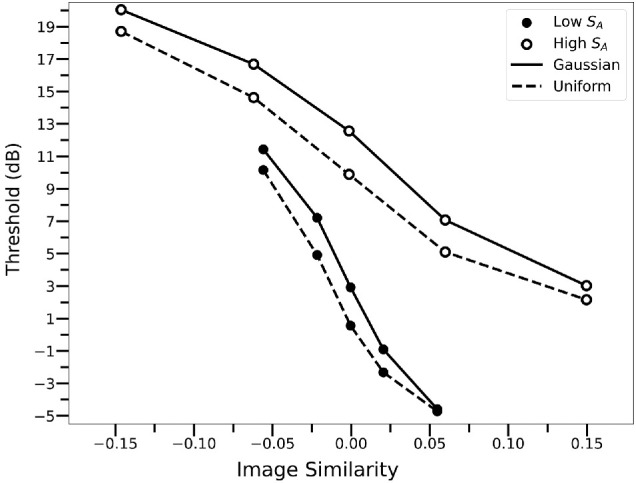
Thresholds of the UETM observer that uses the sum rule rather than the max rule. The thresholds are nearly indistinguishable between the two rules (compare with Figure 4D).

When we simulated the UETM observer using the sum rule (Equation 7), we found that it predicts thresholds very similar to those predicted for the max rule (see Appendix Figure A2). Thus, the pattern of human thresholds does not discriminate between the two rules. This result is also intuitive because the sum is dominated by the max and the template responses from locations near the max.

**Figure A3.**
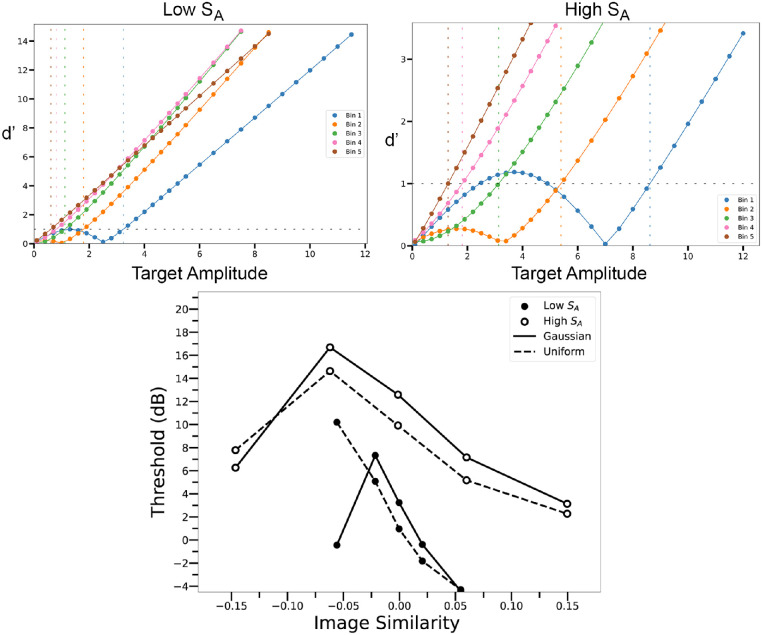
Psychometric functions and thresholds for the UETM observer under blocked conditions.

If the presentations in the different image similarity bins were blocked then, in the low percentile trials, it might have been possible for the subjects to estimate that the image similarity is negative (the background is out of phase with the target) and detect the target as a reduction in the template response (a reduction in contrast). However, this is unlikely in the current experiment where only the target amplitude and the amplitude-spectrum similarity were blocked. Indeed, if subjects were only looking for the max or sum, then we would expect negative *d*′ values (*PC*_max_ < 0.5) for low target amplitudes in the most negative image-similarity bins, which is what we observed (see Figure 3). We have simulated performance of the max and sum rules when the model observer knows, for each amplitude level, whether the distribution of template responses when the target is present, is to the right or to the left compared to that when the target is absent (see Appendix Figure A3). Even in this case, the predicted masking functions remain asymmetric, except for a decrease in threshold for the lowest image similarity bin.

#### Observers with a mixture of simple and complex templates

**Figure 5.**
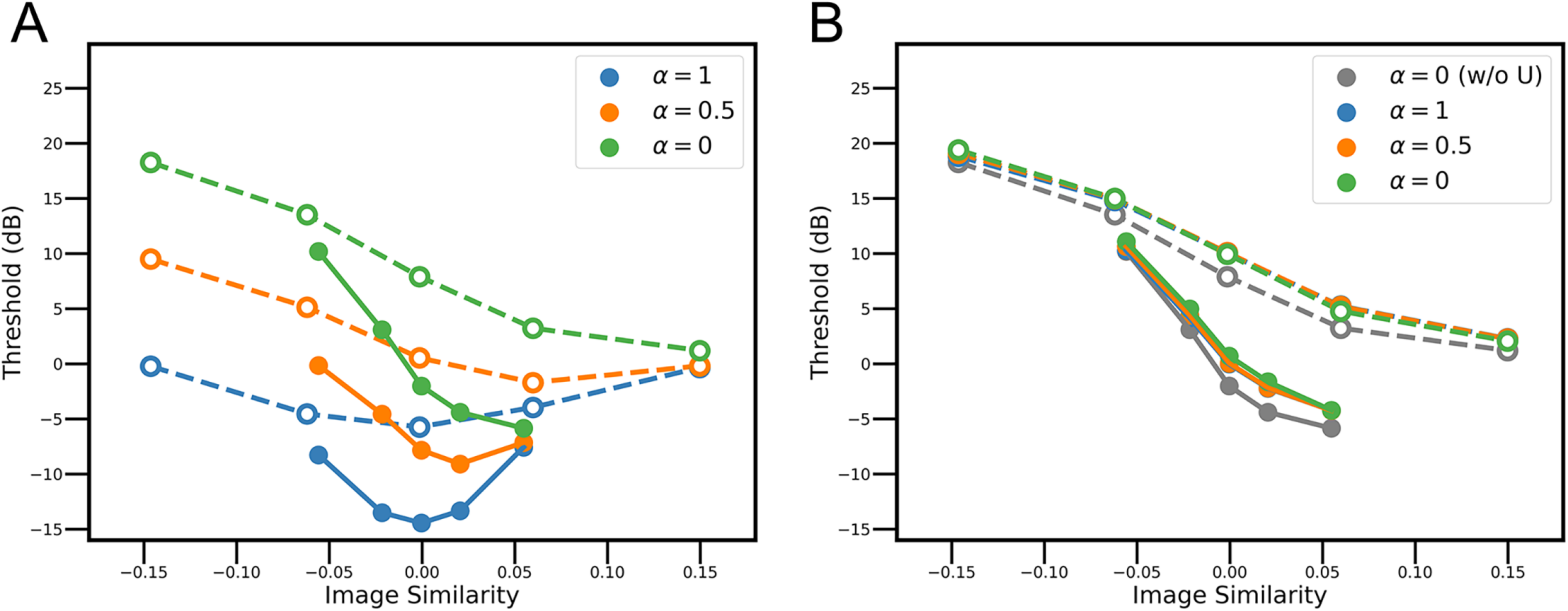
Predictions for mixtures of simple and complex templates. (**A**) Thresholds of the ETM observer that includes simple and/or complex template responses. (**B**) Thresholds of the UETM observer that includes simple and/or complex template responses. α = 1: only simple template response, α = 0.5: equal weight on simple and complex template responses, α = 0: only complex template response. The gray curves in B are a replot of the green curves in A.

Figure 5 [Fig figA1] [Fig figA2] shows the effect of incorporating complex templates into model observers both with and without position uncertainty. The plots in Figure 5 are only for models that include the human CSF (eye filter). Figure 5A shows the model thresholds when there is no position uncertainty, for mixture parameter values of 1.0, 0.5, and 0.0. When the mixture parameter is 1.0, there are only simple template responses and hence the model thresholds are identical to those in Figure 4B. When the mixture parameter is 0.0 there are only complex template responses. In this case, the thresholds are highly asymmetrical, even though there is no position uncertainty. When the mixture parameter is 0.5 the thresholds are asymmetrical, but less so than when the mixture parameter is 0.0.

Figure 5B shows the thresholds when uniform position uncertainty is included. When the mixture parameter is 1.0, there are only simple template responses, and, hence, the model thresholds are identical to the dashed curves in Figure 4D. Interestingly, the UETM observer's thresholds are almost completely unaffected by decreasing the mixture parameter. Furthermore, the thresholds when using only the complex template are almost completely unaffected by the intrinsic position uncertainty: the gray curves in Figure 5B replot the green curves in Figure 5A.

These seemingly puzzling results can be explained by the fact that a complex template effectively sums energy over the template region independent of the spatial phase. In other words, the complex template is implementing an approximate sum rule (or max rule) over the template region; thus, applying the sum or max rule to the complex template responses over the uncertainty region has little additional effect. The fact that both simple and complex templates perform equally well under our experimental conditions suggests even in tasks like ours, where it would be best to suppress energy at irrelevant phases, it could still be beneficial to pool over simple and complex cell responses. If there were no position uncertainty, we would expect the visual system to strongly down weight the complex cell responses. These modeling results (Figure 5B) also show that the predicted effects of image similarity are robust to the relative contributions of simple and complex templates (cells).

## Discussion

In previous studies, we measured the effect of various properties of natural backgrounds on the detectability of specific targets, presented in the fovea, for the duration of a typical fixation during visual search. One of these dimensions was the cosine similarity of the amplitude spectrum of the background to that of the target, which is a phase-invariant measure of similarity. Here, we measured the effect of the cosine similarity between the images of the background and target, while holding the phase-invariant similarity at fixed levels. We find that the phase-invariant and phase-dependent similarities both have a substantial effect on target detectability, and hence both are important factors to include in theories of visual detection and search in natural backgrounds.

As in previous studies, we used template-matching observers to generate predictions for the different experimental conditions, because they exploit most of the available information and hence provide a reasonable benchmark. The thresholds of template-matching observers are a linear function of amplitude spectrum similarity, like the thresholds of human observers. Also, the linear relationship holds even when intrinsic position uncertainty is included in the template-matching observer, although thresholds are scaled up by a factor that depends on the level of uncertainty (Walshe & Geisler, 2020).

The story is very different for image similarity. Human thresholds decline monotonically with the level of image similarity, whereas the template-matching observer's threshold is a U-shaped function of similarity with a minimum when target and background are orthogonal. This is a striking difference. However, when we include the small level of human intrinsic position uncertainty measured in a separate experiment, we find that the template-matching observer now produces a similar pattern of thresholds to the human observers. Thus intrinsic position uncertainty has a rather complex effect on detection in natural backgrounds.

The UETM observer uses the average human CSF (Watson & Ahumada, 2005) and the level of intrinsic position uncertainty estimated directly for our stimulus conditions, and thus has no free parameters. Although the predictions (Figure 4D) are qualitatively similar to the human thresholds (Figure 4A), there are quantitative differences. Specifically, the predicted effect of image similarity is larger than the observed effect. We have considered several plausible potential explanations. We considered including templates that pool over phase (like complex cells) in addition to templates that do not (like simple cells) but found that it does not change the predicted size of the effect (Figure 5). We also considered the effect of using incomplete templates (i.e., ignoring half of the pixels in the target) and also found that it produces almost no change in the size of the effect. Another obvious factor is internal noise. Double-pass experiments suggest that internal noise level is proportional to the external noise level (Burgess & Colborne, 1988). Plausible values of the proportionality constant reduce the predicted size of the effect from about 16 dB to about 10 dB. The measured size of the effect is about 6-7 dB; thus, at this point we do not have an entirely satisfactory explanation for why the effects of image similarity are smaller than predicted.

The current study was motivated by a recent study (Rideaux et al., 2022) that measured accuracy in a covert search task, where a derivative-of-Gaussian target was added to a single location (or to multiple locations) in a 2^o^ diameter natural image. On each trial, two different natural images were presented for 200 ms on either side of fixation, and the subjects reported which image contained at least one target. They found that accuracy was higher when the targets were in phase with the background. They also found that amplitude spectrum similarity had a weaker effect. Their study differed in several ways from the current study, which could explain the differences. First, their task included substantial extrinsic position uncertainty (the targets could appear anywhere within the two 2^o^ backgrounds), which was much bigger than the intrinsic position uncertainty measured in the current study. Second, the backgrounds were images of “things” and not natural outdoor scenes like those in the current study. Third, their target locations were picked in a more constrained fashion by simultaneously searching the background and varying the orientation of the target to find the best match. Their target locations were also constrained to be at the highest contrast locations. Nonetheless, their results are consistent with our hypothesis that image similarity and position uncertainty combine to produce highly asymmetric masking effects in natural backgrounds.

The current study together with our previous studies (Sebastian et al., 2017; Sebastian et al., 2020; Zhang & Geisler, 2022) provide strong evidence that there are at least five distinct stimulus factors and four known biological factors that that strongly contribute to detection performance for localized additive targets in achromatic natural backgrounds.

**Figure A4.**
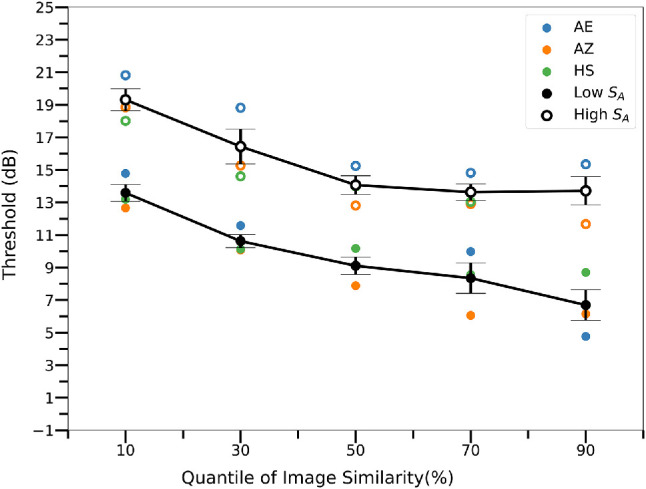
The thresholds in Figure 4A replotted as a function of the quantile of image similarity.

The five stimulus factors are the local background luminance (*L*), RMS contrast (*C*), partial-masking factor (‖*T_p_*‖), amplitude-spectrum similarity (*S_A_*), and image similarity (*S*_*I*|*A*_). For the targets we have tested so far, the first four of these factors combine approximately multiplicatively: amplitude threshold is proportional to the product of luminance, contrast, amplitude-spectrum similarity, and inverse partial-masking factor. Image similarity also appears to be a distinct factor, but it is not yet clear how it combines with the other stimulus factors. One hint of regularity is revealed by plotting the threshold in dB as a function of the percentile image similarity rather than image similarity itself. When plotted in these units the threshold curves are fairly parallel (see Appendix Figure A4), consistent with a separable interaction between the effect of amplitude-spectrum similarity and percentile image similarity. How general is this result remains to be seen.

**Figure A5.**
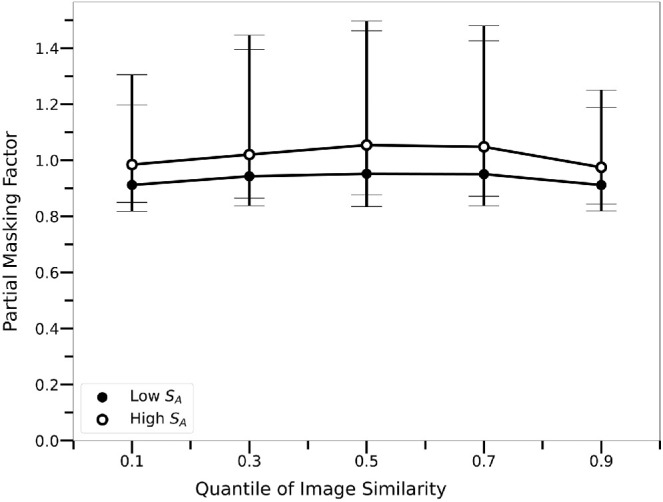
Partial-masking factors for the natural-background bins in the current study.

Another question is whether the effect of image similarity is independent of the partial-masking factor, which was not controlled in the current study. In the Appendix, we show that the partial-masking factor was nearly constant across all levels of image similarity, consistent with the independence hypothesis (see Appendix Figure A5).

The four biological factors are the CSF (optics and retinal center-surround effects), intrinsic position uncertainty, normalization, and foveation (the variation in spatial resolution with retinal location). The first two of these factors were directly considered in the current study, with intrinsic position uncertainty having a rather surprising effect on performance. Intrinsic position uncertainty is likely to be a fairly complex factor. First of all, it depends on the reference frame (context) for the target location estimates. For the 4.3^o^ diameter patches in the current task, the position discrimination thresholds at the center of the reference frame are approximately 2 arcmin, but they will vary with the diameter of the reference frame (Legge & Campbell, 1981) and are likely to also vary with the shape of the frame and the location within the frame. Second, intrinsic position uncertainty increases substantially with retinal eccentricity (Michel & Geisler, 2011). An important direction for future research would be to develop a theory that can predict the magnitude of intrinsic position uncertainty for arbitrary reference frames/contexts. In the meantime, intrinsic position uncertainty can be directly estimated from position-discrimination psychometric functions measured for the reference frames in a given detection experiment.

In the current study, detection psychometric functions were measured in the center of the fovea with a different natural background presented on every trial, from the same amplitude-spectrum-similarity bin. In addition to blocking by amplitude-spectrum similarity, the target amplitude was fixed within each sub-block, and was presented with a prior probability of 0.5. Under these circumstances (without intrinsic position uncertainty) good performance can be obtained by placing a single fixed criterion on the template-response axis. However, under more natural conditions, where the target-present prior is low and the background properties are random (not blocked), good performance cannot be achieved with a single fixed criterion unless the template responses are first normalized by the luminance, contrast, and amplitude spectrum similarity in the target region (Sebastian et al., 2017; Sebastian et al., 2020). Such normalization occurs in the early visual system and is critical for good performance under real-world conditions.

The final factor is foveation. A general theory of target detectability in natural backgrounds must take into account, at the least, how the CSF, intrinsic position uncertainty, and normalization vary with retinal location.

## Appendix

Here we provide additional data analyses and simulations that support some of the claims made in the results and discussion. We also provide a formal derivation of the sum and max rules in the Bayesian observer framework.

### Performance of UETM observer for blocked conditions

The upper plots in Figure A3 show the dʹ psychometric functions of the UETM observer, for the max rule and the uniform intrinsic-uncertainty prior, under the assumption that image similarity, amplitude-spectrum similarity, and target amplitude are fixed within blocks of trials. Under these conditions, the observer knows (or can learn) whether the mean max-template response is larger or smaller in the target-present trials than in the target-absent trials, and can adjust the decision rule accordingly. As can be seen, there are only positive values of dʹ; accuracy never falls below 50%, while human observers had negative dʹ and below-chance accuracy (see Figure 3). The lower plot in Figure A3 shows the predicted thresholds. The pattern of results is similar to when the image similarity is unblocked (Figure 4D), except for the decline in threshold when the image similarity is lowest, and the amplitude spectrum similarity is highest. Note that if the criterion defining threshold were higher (e.g., dʹ = 1.5) then the predicted thresholds would decrease monotonically.

### Independent effects of different factors

Figure A4 is a replot of Figure 4A with image similarity expressed in units of percentile rather than the specific bin value. In these units the curves are approximately parallel. Whether this systematic relationship holds for other levels of amplitude-spectrum similarity remains to be seen.

Figure A5 plots the mean and 67% confidence interval of the partial-masking factor for the five image similarity bins and the two amplitude-spectrum similarity bins. The mean partial-masking factor is nearly constant across the natural-background bins in the current study.

### Derivation of sum and max rules

Here we provide a formal derivation of the max and sum rules. We begin by deriving the general case, which only assumes statistical independence of the responses at the potential target locations. We assume there are *n* potential target locations and that the target may be absent or may appear at any one of the *n* locations with some prior probability *p*(*i*), where *i* indexes a potential target location. The prior probability of target absent is represented by *p*(0); in other words, *i* can range from 0 to *n*, where the sum of the prior probabilities from 0 to *n* equals 1.0. On each trial, a vector of responses **R**_*i*_ is produced at each potential target location. The probability distribution of this vector of responses is arbitrary (e.g., need not be Gaussian), but the responses at the potential locations are assumed to be statistically independent across potential locations and trials.

Consider first the sum rule, which is the optimal decision rule for independent locations when the goal is maximize the observer's accuracy in reporting whether the target is present or absent (the task does not include reporting the target's location). The maximum accuracy is obtained by computing the probability that the target is absent given the observed responses at the potential target locations. Using Bayes rule we have:

(1A) $$\begin{array}{ccc} & & \;p\left(0\left|R_{1},\cdots ,R_{n}\right.\right)\\ & & \;=\frac{p\left(R_{1},\cdots ,R_{n}\left|0\right.\right)p\left(0\right)}{p\left(R_{1},\cdots ,R_{n}\left|0\right.\right)p\left(0\right)+p\left(R_{1},\cdots ,R_{n}\left|\neg 0\right.\right)\left(1-p\left(0\right)\right)}\; \end{array}$$

or equivalently,

(2A) $$\begin{array}{c} p\left(0\left|R_{1},\cdots ,R_{n}\right.\right)=\frac{1}{1+\frac{p\left(R_{1},\cdots ,R_{n}\left|\neg 0\right.\right)\left(1-p\left(0\right)\right)}{p\left(R_{1},\cdots ,R_{n}\left|0\right.\right)p\left(0\right)}}\; \end{array}$$

Thus the decision rule that maximizes accuracy is to report target absent if

(3A) $$\begin{array}{c} \frac{p\left(R_{1},\cdots ,R_{n}\left|\neg 0\right.\right)}{p\left(R_{1},\cdots ,R_{n}\left|0\right.\right)}<\frac{p\left(0\right)}{1-p\left(0\right)}\; \end{array}$$

Given statistical independence, the decision rule becomes

$$\begin{array}{c} \sum_{j=1}^{n}p\left(j\right)\prod_{i=1}^{n}\frac{p\left(R_{i}\left|\neg 0,j\right.\right)}{p\left(R_{i}\left|0\right.\right)}<\frac{p\left(0\right)}{1-p\left(0\right)} \end{array}$$

All the ratios in the product where *i* ≠ *j* cancel, and, hence, the decision rule is “target present” if

(4A) $$\begin{array}{c} \sum_{j=1}^{n}p\left(j\right)L_{j}>\frac{p\left(0\right)}{1-p\left(0\right)}\; \end{array}$$

where *L_i_* = *p*(**R**_*i*_|*j*)/*p*(**R**_*i*_|0) is the likelihood ratio for the response observed at location *i*, given the target is at location *j* versus absent.

Next consider the max rule, which is the optimal decision rule for independent locations when the goal is to maximize the observer's accuracy in reporting either the location of the target or that the target is absent. If we regard target absent as location *j* = 0, then accuracy will be maximized by simply picking the location with the highest posterior probability. Using Bayes rule the posterior probabilities are given by

(5A) $$\begin{array}{ccc} & & \;p\left(i\left|R_{1},\cdots ,R_{n}\right.\right)=\frac{p\left(R_{1},\cdots ,R_{n}\left|i\right.\right)p\left(i\right)}{\sum_{j=0}^{n}p\left(R_{1},\cdots ,R_{n}\left|j\right.\right)p\left(j\right)}\; \end{array}$$

or,

$$\begin{array}{c} p\left(i\left|R_{1},\cdots ,R_{n}\right.\right)=\frac{1}{\sum_{j=0}^{n}\frac{p\left(R_{1},\cdots ,R_{n}\left|j\right.\right)p\left(j\right)}{p\left(R_{1},\cdots ,R_{n}\left|i\right.\right)p\left(i\right)}} \end{array}$$

Assuming statistical independence and cancelling terms that are neither *i* nor *j* we have

(6A) $$\begin{array}{c} p\left(i\left|R_{1},\cdots ,R_{n}\right.\right)=\frac{1}{1+\sum_{j\neq i}\frac{p_{j}}{p_{i}}\frac{p\left[R_{i}\left|j\right.\right]p\left[R_{j}\left|j\right.\right]}{p\left[R_{i}\left|i\right.\right]p\left[R_{j}\left|i\right.\right]}}\; \end{array}$$

Let

$$\begin{array}{c} X_{ij}=\frac{p_{j}}{p_{i}}\frac{p\left[R_{i}\left|j\right.\right]p\left[R_{j}\left|j\right.\right]}{p\left[R_{i}\left|i\right.\right]p\left[R_{j}\left|i\right.\right]} \end{array}$$

then

$$\begin{array}{c} p\left(i\left|R_{1},\cdots ,R_{n}\right.\right)=\frac{1}{1+\sum_{j\neq i}X_{ij}} \end{array}$$

Now, let

(7A) $$\begin{array}{c} Y_{i}=\frac{p_{i}}{p_{0}}\frac{p\left[R_{j}\left|i\right.\right]p\left[R_{i}\left|i\right.\right]}{p\left[R_{j}\left|0\right.\right]p\left[R_{i}\left|0\right.\right]}=\frac{p_{i}}{p_{0}}\frac{p\left[R_{i}\left|i\right.\right]}{p\left[R_{i}\left|0\right.\right]}\; \end{array}$$

where *i* ≠ *j*. It follows that

(8A) $$\begin{array}{c} X_{ij}=\frac{p_{j}}{p_{i}}\frac{p\left[R_{i}\left|j\right.\right]p\left[R_{j}\left|j\right.\right]}{p\left[R_{i}\left|i\right.\right]p\left[R_{j}\left|i\right.\right]}=\frac{Y_{j}}{Y_{i}}\; \end{array}$$

And, hence,

(9A) $$\begin{array}{ccc} & & \;p\left(i\left|R_{1},\cdots ,R_{n}\right.\right)\\ & & \;\;\;\;=\frac{1}{1+\sum_{j\neq i}X_{ij}}=\frac{Y_{i}}{1+\sum_{i=1}^{n}Y_{i}}=\frac{p_{i}L_{i}}{\sum_{i=0}^{n}p_{i}L_{i}}\; \end{array}$$

The denominators for all the posterior probabilities are the same, and thus the optimal response *i_opt_* is given by

(10A) $$\begin{array}{ccc} & & \;i_{opt}=\underset{i}{\arg\max}\left(p_{i}L_{i}\right)=\underset{i}{\arg\max}\left(\ln p_{i}+\ln L_{i}\right)\; \end{array}$$

where *L*_0_ = 1.

Now consider the special cases of the sum and max rules used in the current study in which the response at each location is a single template response, and where we assume that the responses are Gaussian distributed, which we have shown they are to good approximation (Sebastian et al., 2017). Let *R_i_* be the unnormalized template response at location *i* to the mean-subtracted image patch at location *i*. The likelihood ratio of the response is

$$\begin{array}{ccc} & & L_{i}=\exp\left(-\frac{{\left(R_{i}-u\right)}^{2}-{\left(R_{i}-u_{0}\right)}^{2}}{2\sigma^{2}}\right)\\ & & \;=\exp\left(\frac{\left(u-u_{0}\right)}{\sigma }\frac{R_{i}}{\sigma }-\frac{u^{2}-u_{0}^{2}}{2\sigma^{2}}\right) \end{array}$$

where *u* and *u*_0_ are mean template response to target present and absent and σ is the standard deviation of the template response. Now, d'=(u-u0)/(u-u0)σσ, the normalized response is Ri/Riσσ, and (u2-u02)/(u2-u02)2σ22σ2 is a constant. Substituting into Equation 10A,

(11A) $$\begin{array}{c} i_{opt}=\underset{i}{\arg\max}\left(\ln p_{i}+d^{'}{R^{'}}_{i}\right)\; \end{array}$$

which agrees with Equation 6. Substituting into Equation 4A gives

(12A) $$\begin{array}{ccc} & & \;\exp\left(-\frac{u^{2}-u_{0}^{2}}{2\sigma^{2}}\right)\sum_{j=1}^{n}p\left(j\right)\exp\left(d^{'}{R^{'}}_{i}\right)>\frac{p\left(0\right)}{1-p\left(0\right)}\; \end{array}$$

which agrees with Equation 7, up to a scalar that only affects the value of the criterion (which we varied to optimize performance).
